# Genome-Wide Association Analyses Identify QTL Hotspots for Yield and Component Traits in Durum Wheat Grown under Yield Potential, Drought, and Heat Stress Environments

**DOI:** 10.3389/fpls.2018.00081

**Published:** 2018-02-06

**Authors:** Sivakumar Sukumaran, Matthew P. Reynolds, Carolina Sansaloni

**Affiliations:** ^1^Global Wheat Program, International Maize and Wheat Improvement Center (CIMMYT), Texcoco, Mexico; ^2^Genetic Resources Program, International Maize and Wheat Improvement Center (CIMMYT), Texcoco, Mexico

**Keywords:** durum, GWAS, heat stress, drought stress, yield potential, molecular markers, QTL hotspots, population structure

## Abstract

Understanding the genetic bases of economically important traits is fundamentally important in enhancing genetic gains in durum wheat. In this study, a durum panel of 208 lines (comprised of elite materials and exotics from the International Maize and Wheat Improvement Center gene bank) were subjected to genome wide association study (GWAS) using 6,211 DArTseq single nucleotide polymorphisms (SNPs). The panel was phenotyped under yield potential (YP), drought stress (DT), and heat stress (HT) conditions for 2 years. Mean yield of the panel was reduced by 72% (to 1.64 t/ha) under HT and by 60% (to 2.33 t/ha) under DT, compared to YP (5.79 t/ha). Whereas, the mean yield of the panel under HT was 30% less than under DT. GWAS identified the largest number of significant marker-trait associations on chromosomes 2A and 2B with *p*-values 10^−06^ to 10^−03^ and the markers from the whole study explained 7–25% variation in the traits. Common markers were identified for stress tolerance indices: stress susceptibility index, stress tolerance, and stress tolerance index estimated for the traits under DT (82 cM on 2B) and HT (68 and 83 cM on 3B; 25 cM on 7A). GWAS of irrigated (YP and HT combined), stressed (DT and HT combined), combined analysis of three environments (YP + DT + HT), and its comparison with trait *per se* and stress indices identified QTL hotspots on chromosomes 2A (54–70 cM) and 2B (75–82 cM). This study enhances our knowledge about the molecular markers associated with grain yield and its components under different stress conditions. It identifies several marker-trait associations for further exploration and validation for marker-assisted breeding.

## Introduction

Durum wheat (2*n* = 28, AABB, *Triticum turgidum* L. ssp. *durum*) is the most commonly cultivated form of allotetraploid wheat, and is grown on 8% of the world's wheat area (FAOStat, 2016). It originated in the Mediterranean region, and is used to make pasta and semolina products (Ren et al., 2013). Approximately 75% of durum wheat is still grown in the Mediterranean basin in irrigated and rainfed environments, which contributes to 50% of the worldwide production (Li et al., 2013; Kabbaj et al., 2017). Climate change will significantly impact the Mediterranean region; temperatures are predicted to increase by 3–5°C, and annual precipitation is likely to decrease by 4–27% during the cropping season (Flato et al., 2013). The frequency and duration of dry spells and heat waves are also likely to increase in dryland areas (Parry et al., 2005; Bates et al., 2008).

Terminal drought and heat stresses negatively affect wheat grain weight and yield (Araus et al., 2003; Slafer et al., 2005). Dissecting the genetic bases of durum wheat responses to drought and heat stress is a prerequisite for breeding future genotypes (Graziani et al., 2014), and can be accomplished through the complementary approaches of association mapping and QTL mapping (Zhu et al., 2008). Association mapping, which is based on linkage disequilibrium (LD), is a powerful approach to genetic mapping that provides high resolution of detected loci, due to the presence of high genetic diversity and historic recombination of alleles in the assembled association mapping population (Sukumaran and Yu, 2014). A new multi-parent approach to map quantitative trait locus (QTL)—Multi-parent advanced generation inter-cross (MAGIC)—is an alternative to traditional linkage mapping but has less genetic diversity than the diverse association mapping panel (Milner et al., 2016). Several association mapping studies have been conducted to dissect the genetic basis of grain yield in durum wheat (Mengistu et al., 2016; Kidane et al., 2017). Maccaferri et al. (2011) evaluated a collection of 189 elite durum wheat lines in 15 environments with varying water availability during the cropping cycle. They identified 56 markers that explained 3.5–4.2% of the variation in grain yield, but the number of marker-trait associations (MTA) under drought stress were less in number compared with irrigated conditions. Several studies have focused on genetic diversity and molecular characterization of durum wheat landraces using different markers systems, but not many have used the DArTseq marker system in durum wheat (Yildirim et al., 2011; Kabbaj et al., 2017; Monostori et al., 2017).

Genetic improvement of durum wheat for drought and heat stresses can be achieved by direct or indirect selection for yield in target environments, or environments like target environments. Direct selection involves selecting for yield, whereas indirect selection—i.e., physiological breeding—selects for yield components or other associated traits such as canopy temperature, normalized difference vegetation index, grain number, and thousand grain weight (TGW; Araus et al., 2008; Reynolds et al., 2009b; Tuberosa, 2012; Reynolds and Langridge, 2016). The difficulty is in knowing which trait combinations should be selected to produce stable high yielding genotypes under varying environmental stress conditions (Habash et al., 2009). It is therefore essential to understand the genetics and gene action of these traits. This study phenotyped a durum panel under yield potential (YP), drought stress (DT), and heat stress (HT) conditions. We studied the genetic diversity of the panel and identified molecular markers associated with grain yield and its components under YP, DT, and HT. In addition, common genomic regions (QTL hotspots) associated with grain yield (YLD), TGW, and grain number m^−2^ (GNO) under YP and DT, and markers for stress indices under DT and HT, were identified.

## Materials and methods

### Germplasm

The 208-line durum wheat panel used in this study is a subset of the 15,000 CIMMYT gene bank accessions that were previously evaluated and characterized for use in breeding for heat and drought tolerance. The panel consisted of durum lines from different International Wheat Improvement Network (IWIN) nurseries: 2IDYN, 3IDYN, 15IDYN, 33EDUYT, 34IDSN, and 24EDYT-SA (Table S1). The lines originated from Ethiopia, Lebanon, Iran, Chile, Mexico, Syria, and Ecuador, and 180 of the 208 crosses were derived at CIMMYT, Mexico, as per the International Wheat Information System (IWIS; http://hdl.handle.net/10568/48661) records.

### Phenotyping

Phenotyping was conducted at the Campo Experimental Norman E Borlaug in Cd. Obregon, Sonora, Mexico, under YP, DT, and HT conditions. Planting date and irrigation schedule were managed to create different stress conditions (Table 1). The experiment was planted in an alpha lattice design with two replications, 2 m plots, 2 rows per plot in raised bed system 75 cm wide, with a 5 g m^−2^ seed planting density, and flooded irrigation in critical developmental stages. The genotypes were arranged in different “blocks” for alpha lattice design based on days to flowering. Irrigation frequency was the same under YP and HT to avoid the confounding effect of drought under HT conditions. N application was dependent on moisture availability and varied from 150 to 200 kg ha^−1^. Fungicides and pesticides were applied to control local diseases and pests. Over two cropping seasons (2014-15 and 2015-16), the following traits were measured in accordance with established protocols (Pask et al., 2012): YLD, GNO, TGW, days to heading (DTH; under HT), days to anthesis (DTA; under YP and DT), days to maturity (DTM), plant height (PH), and normalized difference vegetation index (NDVI) at vegetative (NDVIvg), and grain filling stages (NDVIllg). Based on our previous research (data not shown), DTH and DTA are highly correlated under YP and DT conditions, but DTH and DTA might occur at the same time under HT conditions, which makes it difficult to measure DTA under HT.

**Table 1:** The durum wheat panel grown under yield potential (YP), drought stress (DT), and heat stress (HT) conditions.

**Year**	**Env**.	**Planting date**	**Harvest date**	**T_mean_**	**T_Range_**	**Prec**.	**Irrig**.	**T_max_ > 35**
2014-15	YP	28-Nov-14	22-May-15	19.4	11.8–28.2	84.2	3	0
	DT	09-Dec-14	07-May-15	19.4	11.8–28.1	84.0	0	0
	HT	20-Mar-15	14-Jul-15	25.9	18.0–34.2	61.2	6	47
2015-16	YP	16-Dec-15	17-May-16	16.1	08.1–26.2	20.4	3	0
	DT	02-Dec-15	13-Apr-16	16.2	08.2–26.4	20.2	0	0
	HT	26-Feb-16	14-Jun-16	22.4	13.4–32.1	17.4	6	26

*Env., environments; T_mean_, Mean temperature during the crop cycle; T_Range_, mean minimum and maximum temperatures; Prec., precipitation; Irrig., No. of irrigations; T_max_ > 35, Number of days that had temperatures above 35°C*.

### High-throughput genotyping

A modified CTAB (cetyltrimethylammonium bromide) method (Saghai-Maroof et al., 1984) was used to extract genomic DNA from fresh leaves collected from the 208 entries. DNA quality and concentration were determined by electrophoresis in 1% agarose gel. High-throughput genotyping was conducted in 96 plex using DArTseqTM technology (Sansaloni et al., 2011) in the Genetic Analysis Service for Agriculture facility at CIMMYT, Mexico. A genomic representation of the samples was generated by digesting the genomic DNA with a combination of two restriction enzymes—*Pst*I (CTGCAG) and *Hpa*II (CCGG)—and ligating barcoded adapters to identify each sample to run within a single lane on the Illumina HiSeq2500 instrument (Illumina Inc., San Diego, CA). Successfully amplified fragments were sequenced up to 77 bases, generating ~500,000 unique reads per sample. A proprietary analytical pipeline developed by DArT P/L was used to generate two types of data, (i) scores for “presence/absence” (dominant) markers, called SilicoDArTs and (ii) SNPs in fragments. A set of filtering parameters were then applied to select high-quality markers for this specific study. To obtain the physical positions of the corresponding DArTseq markers, the sequences of the DNA fragments were BLASTed against a local database containing the wheat consensus map v.4 (diversityarrays.com) and to the wheat reference genome sequence from International Wheat Genome Sequencing Consortium (IWGSC) WGA v0.4 (NRGene DeNovoMAGIC), with expected values (E) <e^10^ and minimum base identity >90%. Sequences of the genome IWGSC WGAv0.4 were obtained from https://urgi.versailles.inra.fr/download/iwgsc/.

### Data availability

The genetic and phenotypic data used in the present study are available at http://hdl.handle.net/11529/11053. We used the consensus map from diversity arrays; physical positions of the markers are available in the above link.

### Linkage disequilibrium (LD)

Understanding the LD pattern in germplasm is important for selecting the marker density required for GWAS and for defining identified QTL regions (Siol et al., 2017). We computed LD decay using the open source R package, “sommer” (Covarrubias-Pazaran, 2016).

### Population structure analysis

Population structure of the durum panel was inferred using the model-based clustering method implemented in STRUCTURE software (Pritchard and Przeworski, 2001; Falush et al., 2007), along with the delta K approach to statistically test the results (Evanno et al., 2005). We used 1,300 random SNPs, at least 5 cM apart, to estimate the population structure. Simulations were run by inferring K from 2 to 10, with 20,000 iterations and 5,000 burn-ins. The results were entered into the structure harvester (http://taylor0.biology.ucla.edu/structureHarvester/) to obtain the panel's delta K statistics (Earl and vonHoldt, 2012). Principal component analyses of the SNP data and neighbor joining tree construction were conducted in MEGA7 software (Kumar et al., 2016) and results from different approaches were compared to deduce population structure.

### Statistical analysis and stress indices

Analyses of variance were performed and best linear unbiased predictions (BLUPs) were obtained using META-R software (Vargas et al., 2013). When estimating BLUPs, genotypes, genotype-by-environment interactions, and environments were considered as random factors, while location, block, and replication were fixed factors. In addition, BLUPs for YLD and other traits were calculated for each treatment using flowering time as a covariate. We also calculated BLUPs for irrigated environments (YP and HT combined), stressed environments (DT and HT combined), and combined analysis of all environments (YP, DT, and HT). Pearson correlation coefficients (*r*) among different traits and locations were calculated using the cor() function in R, and the corrplot() package was used to plot the results. Broad sense repeatability (H^2^) was estimated using:

$$\begin{array}{l} {\text{H}}^{2}=\frac{\sigma_{g}^{2}}{\sigma_{g}^{2}+\sigma_{ge}^{2}/l+\sigma_{e}^{2}/rl} \end{array}$$

where $\;\sigma_{g}^{2}$ is the genotypic variance, $\sigma_{ge}^{2}$ is the genotype by environment interaction variance, $\sigma_{e}^{2}$ is error variance, *r* is the number of replications, and *l* is the number of environments.

### Heat and drought stress indices

Genotypes tolerant to drought and heat stresses were identified using three indices. Firstly, the stress susceptibility index (SSI) = [1 − (Ys)/(Yp)]/[1 − ($\bar{\text{Y}}$s)/($\bar{\text{Y}}$p)], where Ys and Yp are yields of the wheat lines evaluated under stress and non-stress conditions, respectively, and $\bar{\text{Y}}$s and $\bar{\text{Y}}$p are the mean yields of wheat lines evaluated under stress and non-stress conditions, respectively (Fischer and Maurer, 1978). Secondly, stress tolerance (TOL) = Yp − Ys (Hossain et al., 1990), and finally stress tolerance index (STI) = (Yp − Ys)/Yp^2^, which we estimated as the percentage reduction under stress conditions.

### Genome-wide association study (GWAS)

MTA analyses were conducted using the TASSEL 5.2.38 software, where generalized linear models and mixed linear models were fitted using SNP data, population structure matrix (Q), kinship matrix (K), coefficient of parentage matrix, and principal components. Principal components and Q matrix were fitted as fixed effects, while coefficient of parentage matrix and K matrix were fitted as random effects (in different combinations) to estimate the best model for each trait (Yu et al., 2006; Zhang et al., 2010; Sukumaran et al., 2014). Model fitting and the best model for each trait was based on the quantile-quantile (Q-Q) plots (Sukumaran et al., 2012; Sukumaran and Yu, 2014). We used a GWAS threshold of –log(*p*) = 3 to declare significant associations, which was determined based on the Q-Q plots and distribution of *p*-values. The Bonferroni correction has a more stringent threshold but—when tested—it did not result in many significant MTAs, so it was not followed.

### Comparison of MTAs and loci through blast searches

We compared significant MTAs to detect association patterns, common loci and unique loci for environments and traits, traits *per se*, and stress indices. We also conducted comparative analyses of the most significant loci with the bread wheat genome assembly TGACv1 (*Triticum aestivum*; http://plants.ensembl.org/Triticum_aestivum/Info/Index), and barley genome assembly Hv_IBSC_PGSB_v2 (*Hordeum vulgare*; http://plants.ensembl.org/Hordeum_vulgare/Info/Index) through BLAST searches to identify syntenic regions and candidate genes, if any.

## Results

### Genetic data, population structure, and LD decay

From the raw genetic data of 53,911 markers, we obtained 6,211 SNPs, after removing monomorphic SNPs and checking minor allele frequency (MAF). SNPs with MAF <5% were excluded, as was missing data (SNPs with >20% missing data). The total length of the genetic map was 2,094 cM. The largest chromosomes were 1A (252 cM) and 1B (286 cM), while the smallest was 6B (84 cM). The average distance between SNPs was 0.34 cM. The length of the A genome was 1,087 cM when compared to the B genome of 1,007 cM. Chromosome 2B had the highest number of SNPs (707), while 4B had the lowest (271).

**Figure 1:**
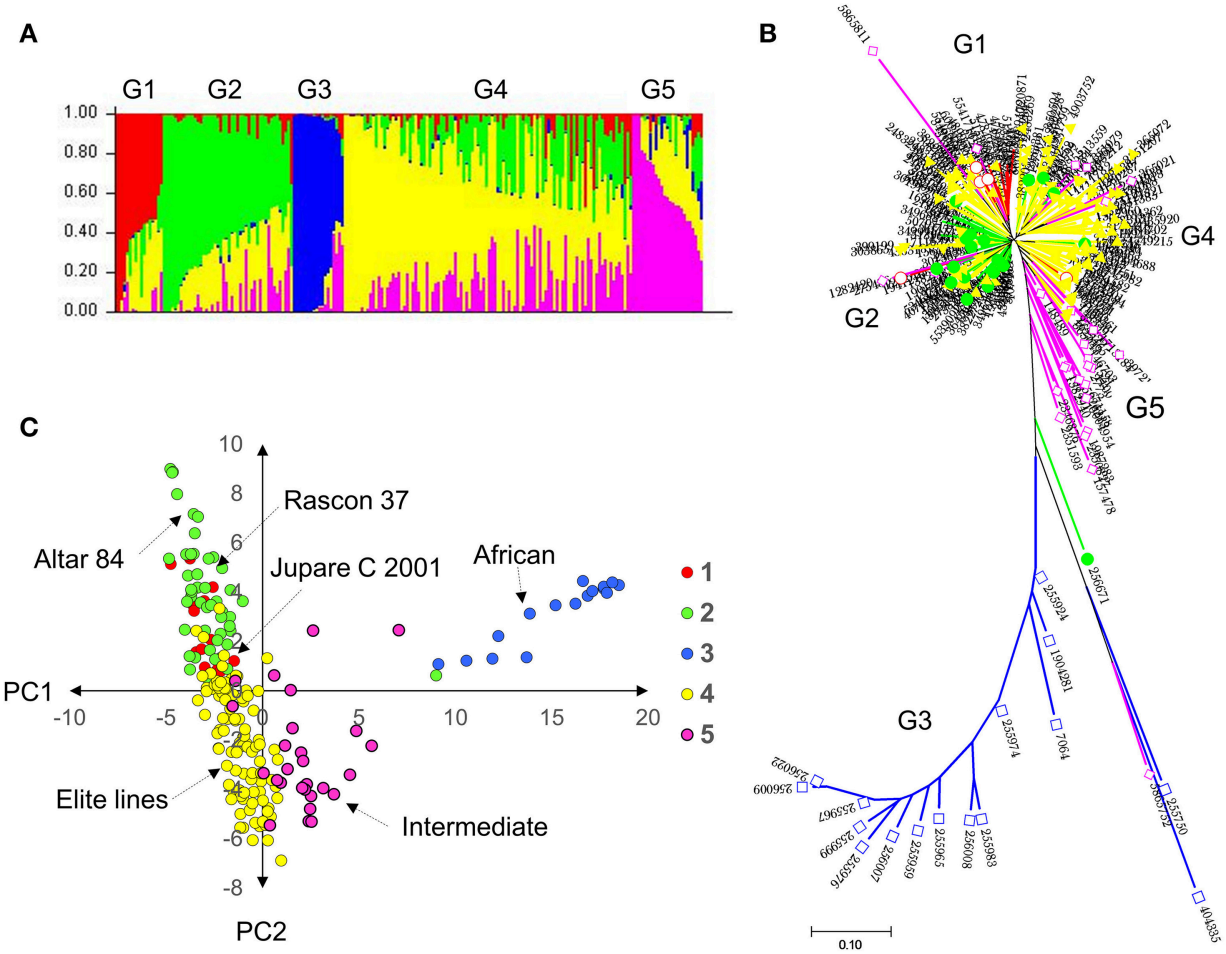
Genetic diversity and population structure of the durum **(A)** probability of population group based on STRUCTURE; **(B)** neighbor joining tree color coded with STRUCTURE probability distribution; and **(C)** principal component analysis based clustering color coded with STRUCTURE results. Results indicate five subpopulations in the durum panel.

Population structure was inferred through different methods. The STRUCTURE algorithm and structure harvester results showed a delta K plot peak value of five (Figure S1). The results from the phylogenetic tree and the principal component analysis plot with principal component (PC)1 vs. PC2, color-coded from the STRUCTURE results, also showed five distinct groups (Figure 1). Group 1 comprised entries with JUPARE C 2001 pedigree, group 2 comprised entries that were crossed to RASCON 37 or ALTAR 84, group 3 comprised entries from Egypt-Africa, group 4 were elite lines derived from several crosses, and group 5 were intermediates between African and elite lines. The group G3 was distant from other groups.

LD analysis used the “sommer” package in R software. The LOESS curve intercepted the line of critical value (*r*^2^ = 2) at 6–8 cM, indicating that—for GWAS—at least one SNP is sufficient within 8 cM region in each chromosome (Figure S2). We observed only for nine gaps >8 cM, which did not affect the GWAS results.

### Agronomic performance of the panel

**Figure 2:**
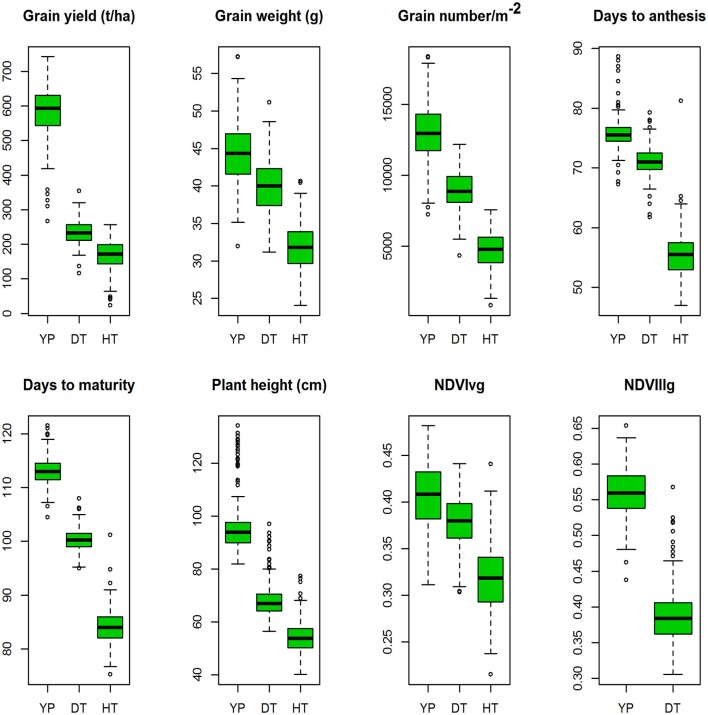
Boxplots of the best linear unbiased predictions (BLUPs) of the traits of the durum panel measured under three different environments in 2014–15 and 2015–16; yield potential (YP), drought stress (DT), and heat stress (HT).

YLD was highest under YP (5.79 t/ha; H^2^ = 0.80), followed by DT (2.33 t/ha; H^2^ = 0.47) and HT (1.64 t/ha; H^2^ = 0.30). TGW also varied among the different environments at 44.4 g (H^2^ = 0.87), 40.8 g (H^2^ = 0.69), and 31.8 g (H^2^ = 0.63), under YP, DT, and HT, respectively. The same trend was observed for all traits (Figure 2). Phenological measurements also followed a similar pattern; the crop had a shorter duration under HT (DTA = 55.5 days; DTM = 84.1 days), compared to DT (DTA = 71 days; DTM = 100 days) and YP (DTA = 76 days; DTM = 113 days). High H^2^*-*values (0.79–0.95) were observed for all traits under YP, except for NDVIvg (H^2^ = 0.30) and NDVIllg (H^2^ = 0.37). Under DT and HT conditions, NDVI values had moderate to high (>0.58) H^2^-values (Table 2). In general, the lowest H^2^-values were observed under HT conditions.

**Table 2:** Descriptive statistics and repeatability (H^2^) estimates by estimating the best linear unbiased predictions (BLUPs) of durum panel grown under yield potential, drought, and heat stress conditions in 2014-15 and 2015-16.

**Traits**	**Yield potential**			**Drought stress**			**Heat stress**		
	**Mean**	**Range**	**H^2^**	**Mean**	**Range**	**H^2^**	**Mean**	**Range**	**H^2^**
YLD^a^	5.79	2.67–7.42	0.80	2.33	1.16–3.54	0.47	1.64	0.2–2.56	0.30
TGW	44.43	31.98–57.24	0.87	40.8	31.1–51.1	0.69	31.8	24.08–40.65	0.63
GNO	12,999	2,090–18,379	0.79	8,914	4,318–12,174	0.22	4,621	811–7,574	0.41
DTA	76	67–102	0.94	71	61–79	0.71	55.5	47–81.2	0.86
DTM	113	104–144	0.90	100	95–108	0.81	84.1	75.5–101.2	0.78
PH	96.8	81.8–134.2	0.95	68.47	56.5–97.1	0.83	54.2	40.1–77.3	0.66
NDVIvg	0.41	0.31–0.48	0.30	0.38	0.30–0.44	0.72	0.32	0.22–0.44	0.58
NDVIllg	0.56	0.44–0.65	0.37	0.39	0.31–0.57	0.82	–	–	–

a *YLD, grain yield (t/ha); TGW, thousand grain weight (g), GNO; grain number/m^2^; DTA, days to anthesis; DTM, days to maturity; PH, plant height (cm); normalized difference vegetative index at vegetative (NDVIvg) and grain filling (NDVIllg) stages*.

**Figure 3:**
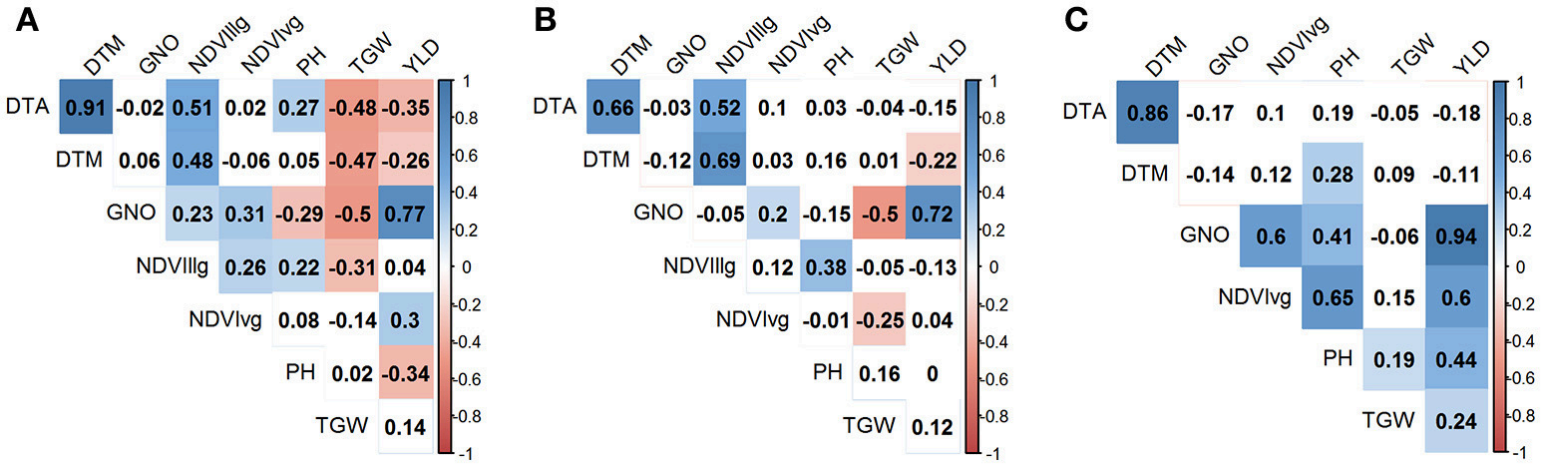
Correlation between the traits (YLD, grain yield (t/ha); TGW, thousand grain weight (g), GNO; grain number/m^2^; DTA, days to anthesis; DTM, days to maturity; PH, plant height (cm); normalized difference vegetative index at vegetative (NDVIvg) and grain filling (NDVIllg) stages) under three different environments **(A)** yield potential; **(B)** drought stress; and **(C)** heat stress. Absolute values > 0.15 were significant at α = 0.05.

Under YP condition, DTA and DTM had the highest correlation coefficient (*r* = 0.91), whereas GNO and YLD were the most correlated traits under DT (*r* = 0.72) and HT (*r* = 0.94). TGW and GNO were negatively associated (*r* = −0.50) under YP and DT, but displayed less pronounced associations (*r* = −0.06, *p*-value not significant) under HT. The association of TGW with YLD was highest under HT (*r* = 0.24), followed by YP (*r* = 0.14), and DT (*r* = 0.12). YLD was negatively associated with DTA (*r* = −0.35), DTM (*r* = −0.26), and PH (*r* = −0.34), under YP, but the effects were not pronounced for YLD vs. DTA under DT (*r* = −0.15) and HT (*r* = −0.18). PH was positively associated with YLD (*r* = 0.44) under HT, but was negatively associated with YLD under YP (*r* = −0.34). NDVIvg was positively associated with all traits under HT conditions, and had the highest association with PH (*r* = 0.65) (Figure 3).

We also estimated 18 stress indices (SSI, TOL, and STI for three traits for two stress environments) and their associations with each other. STI and SSI were negatively associated in several cases (*r* = −1). The highest positive association (*r* = 1) was between TGW SSI vs. TGW STI under HT conditions (Figure S3).

### Marker-trait associations under different environments

#### Yield potential

Under YP, 121 MTAs were identified with *p* < 10^−03^. The trait variation explained by each marker (*R*^2^) varied from 0.07 to 0.11 (Table S2). The highest number of MTAs were identified for PH (30), followed by TGW (25). A comparison of the MTAs identified two loci associated with YLD, TGW, and GNO under YP on chromosomes 2A (54–70 cM) and 2B (78–82 cM). The locus on 2A was not associated with DTA whereas the locus on 2B was associated with DTA, DTM, PH, NDVIvg, and NDVIllg. A locus on chromosome 6A (34 cM) was associated only with YLD, whereas a locus on 5B was associated with PH and YLD, and one on 6B was associated with YLD, DTA, and DTM. DTA and DTM were highly correlated and had several common MTAs, meanwhile NDVIvg and NDVIllg had common MTAs with YLD in chromosome 2B (75–78 cM). MTAs for TGW and GNO that did not affect YLD were identified in chromosomes 5B (82 cM) and 7B (95 cM), respectively (Table S2).

GWAS identified fewer MTAs when DTA was used as a covariate in the BLUPs (compared to without DTA), but the consistent MTAs remained the same. The most consistent region associated with multiple traits was on chromosome 2B (74–82 cM), for YLD, GNO, TGW, DTA, DTM, and PH. The second most consistent region was on chromosome 2A (54–74 cM), for GNO, TGW, DTA, DTM, and PH (Table 3). The highest number of MTAs was for PH, followed by DTM. MTAs for GNO and TGW on chromosome 2A (61–70 cM) were not associated with YLD, potentially indicating a compensation effect (Table S3).

**Table 3:** Comparison of GWAS results for the traits under yield potential conditions using flowering time as a covariate.

**Chr**.	**Traits**							
	**YLD**	**GNO**	**TGW**	**DTA**	**DTM**	**PH**	**NDVIvg**	**NDVIllg**
1A	–	–	–	–	–	84	–	–
1B	–	–	–	6–8, 71, 160	–	142, 269	–	–
2A	–	54–59, 61–67	45, 61–70	23, 33	66, 103	29, 75–76	–	–
2B	74–75	75	75–82	22, 75–80	75–82	63, 78–79	–	–
3A	–	–	–	40	–	1–9	12	–
3B	17	–	–	–	139	49	–	–
4A	–	–	–	–	127–132	–	–	–
4B	–	–	–	2	–	31–39	–	–
5A	–	–	50	–	–		–	–
5B	–	–	77	–	–	63–74	–	–
6A	–	–	–	86	63	58	–	–
6B	–	–	–	23, 68	–	30–35, 47	–	–
7A	40	75	–	–	76, 88, 96	88	–	–
7B	11	36	–	–	–	42, 127–129	–	–

*For each trait, the genetic position is represented by numbers. YLD, grain yield (t/ha); TGW, thousand grain weight (g), GNO; grain number/m^2^; DTA, days to anthesis; DTM, days to maturity; PH, plant height (cm); normalized difference vegetative index at vegetative (NDVIvg) and grain filling (NDVIllg) stages*.

#### Drought stress

Under DT, 159 significant MTAs were identified for eight different traits (Table S4). Of these traits, DTA had the highest number of MTAs (44). Common regions for YLD, TGW, and GNO were not identified under DT, but MTAs for TGW were identified on chromosome 2A (65–70 cM), which was also associated with GNO and NDVIllg. A locus on chromosome 2B (70–82 cM) had significant MTAs for DTM, NDVIllg, DTA, GNO, PH, NDVIvg, and TGW. Meanwhile a locus on chromosome 4A (95–102 cM) was significantly associated with YLD and NDVIvg. MTAs for YLD were identified for six loci, of which five were independent of phenology: chromosome 1A (140–145 cM), 4A (95–96 cM), 5B (30 and 60 cM), 7B (134 cM) (Table S4). Using DTA as a covariate in the calculation for BLUPs and GWAS under DT identified several consistent SNPs (Table S5). The most consistent among them was on 7B (36–40 cM), which was associated with YLD, GNO, and NDVIllg (Table 4). A locus on chromosome 2A (66–70 cM) that was associated with TGW under YP was also associated with TGW under DT. A locus on chromosome 2B (75 cM) was associated with GNO, DTA, PH, and NDVIllg under DT.

**Table 4:** Comparison of the GWAS results for traits under drought stress conditions using days to anthesis as a covariate.

**Chr**.	**Traits**							
	**YLD**	**GNO**	**TGW**	**DTA**	**DTM**	**PH**	**NDVIvg**	**NDVIllg**
1A	140	–	–	–	–	116–117	97	6
1B	99, 223	–	–	142, 160	6	51, 262–271	–	–
2A	–	–	66–70	33, 68–69	–	45	–	–
2B	18	75	–	12, 22, 63–83	–	79, 107	–	78
3A	–	–	69–74	40–48	–	9	–	–
3B	133	–	–	38–49	–	–	60, 64	–
4A	–		–	–	–	–	–	130
4B	–	–	–	1, 32–33	–	31–32	46	–
5A	–	–	–	37, 50, 93	–	–	–	–
5B	–	40	–	42, 55, 82, 89	137–138	65	–	–
6A	54	–	–	28	–	12	–	–
6B	–	–	–	31, 56	1, 68	–	–	68
7A	–	–	–	40	–	88	–	–
7B	39–40	36, 40	–	–	–	59	26	39

*For each trait, the genetic position is represented by numbers. YLD, grain yield (t/ha); TGW, thousand grain weight (g), GNO; grain number/m^2^; DTA, days to anthesis; DTM, days to maturity; PH, plant height (cm); normalized difference vegetative index at vegetative (NDVIvg) and grain filling (NDVIllg) stages*.

#### Heat stress

Under HT, 112 MTAs were detected for the seven traits, excluding NDVIllg. TGW was the trait with highest number of MTAs (61) (Table S6). Eight MTAs were detected for YLD; two of these, on chromosomes 2B (42 cM) and 4A (107–124 cM), were also associated with GNO and YLD. Use of DTA as a covariate showed association of the locus, in chromosome 2B (42 cM), only with YLD not with GNO (Table S7). Another locus on chromosome 4A (124 cM) was also associated with YLD, and with TGW when DTA was not used as a covariate. Twenty loci were associated with DTA (*p* < 0.001), with markers explaining 6–17% of trait variation. An MTA on chromosome 2B (0 cM) explained the most variation among all traits (17%) and was associated with DTM. Another seven MTAs were detected for DTM, of which an MTA on chromosome 5A (40 cM) explained 9% of the variation. For NDVIvg, MTAs were detected on chromosomes 4B and 7A (Table 5). A locus on chromosome 2B (74–82 cM) harbored MTAs for GNO, TGW, and PH (Table 5).

**Table 5:** Comparison of the GWAS results for the traits under heat stress conditions using days to anthesis as a covariate.

**Chr**.	**Traits**						
	**YLD**	**GNO**	**TGW**	**DTA**	**DTM**	**PH**	**NDVIvg**
1A	–	–	–	–	83–85	136	–
1B	–	–	–	6, 8, 71	142	161–162	–
2A	–	–	–	–	–	8	–
2B	42	74–75	81–82	0	63	79	–
3A	–	–	–	69	–	–	–
3B	–	–	–	–	–	–	–
4A	124	–	–	116, 132	–	–	–
4B	–	–	–	–	–	31–32	2
5A	–	65–70	–	–	41, 48	–	–
5B	–	–	–	55, 59	47	–	–
6A	–	–	–	28	–	–	–
6B	–	–	–	–	31	35	–
7A	–	–	–	112	–	96	75
7B	–	–	–	–	–	104	–

*For each trait, the genetic position is represented by numbers. YLD, grain yield (t/ha); TGW, thousand grain weight (g), GNO; grain number/m^2^; DTA, days to anthesis; DTM, days to maturity; PH, plant height (cm); normalized difference vegetative index at vegetative (NDVIvg) and grain filling (NDVIllg) stages*.

### Comparison of MTAs for YLD, GNO, and TGW under different environments

**Figure 4:**
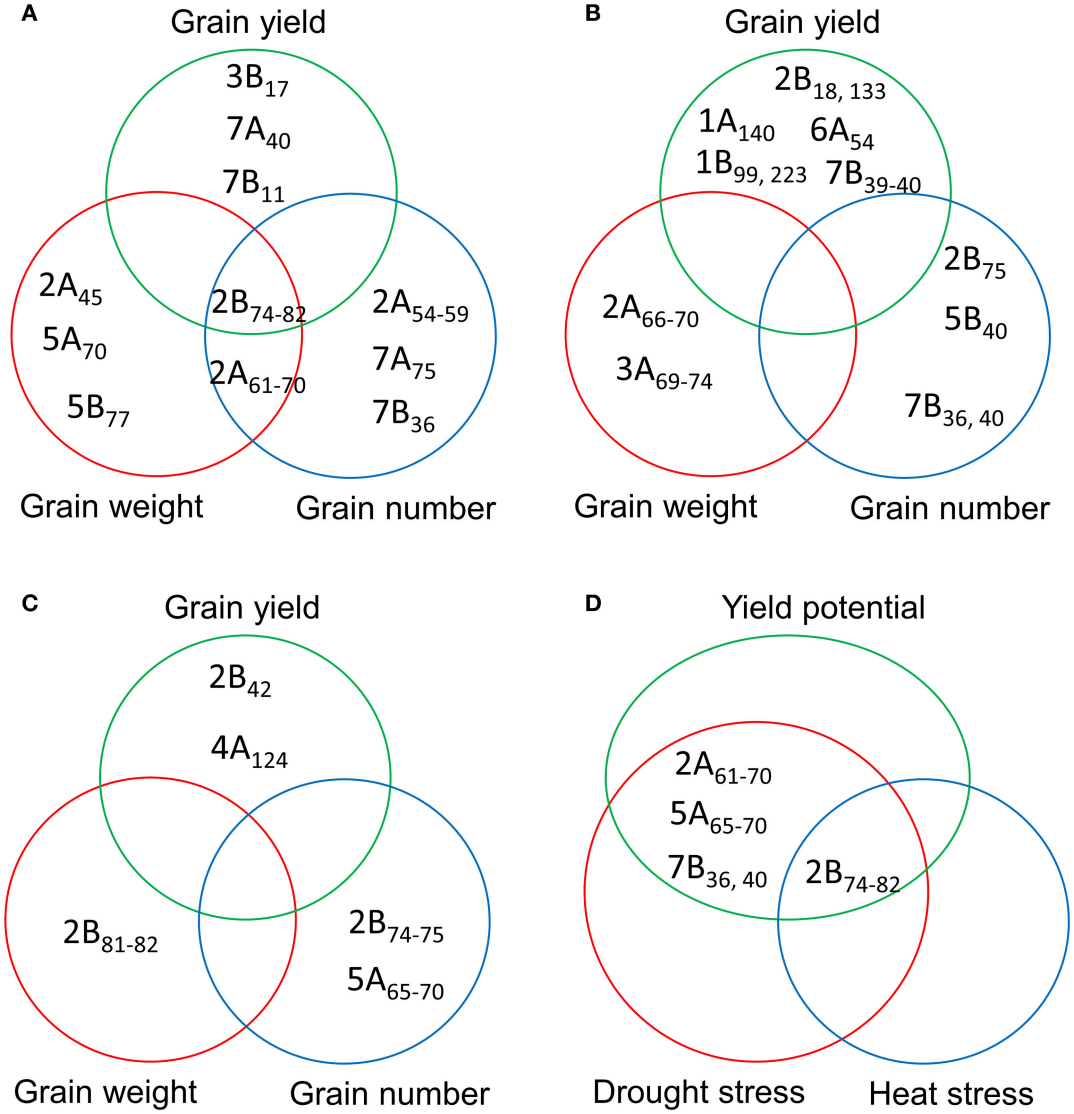
Comparison of the significant marker-trait associations (MTAs) for grain yield, grain number, and thousand-grain weight under **(A)** yield potential; **(B)** drought stress; and **(C)** heat stress conditions; and **(D)** most common MTAs under three different environments.

A comparison of the MTAs for YLD, GNO, and TGW identified a locus in chromosome 2B (74–82 cM) as the most common locus for these three traits under YP. Another locus common to GNO and TGW was located on chromosome 2A (61–70 cM). Several loci for YLD, which were independent of TGW and GNO, were also identified on chromosomes 3B (17 cM), 7A (40 cM), and 7B (11 cM) (Figure 4A). Under DT, no common loci were identified for YLD, GNO, and TGW, but independent loci were identified for all three traits (Figure 4B). Similarly, no common loci were identified under HT, but a locus on chromosome 2B (81–82 cM) was associated with TGW and another at 74–75 cM was associated with GNO (Figure 4C). For all three environments, the most common locus for various traits was on chromosome 2B (74–82 cM) (Figure 4D). These results indicate that this region on chromosome 2B (74–82 cM) harbors genes for TGW and GNO. It is a region that is not in high LD, but is a QTL hotspot.

### Markers for stress tolerance indices

Three different stress tolerance indices (SSI, TOL, and STI) were calculated for YLD, GNO, and TGW by comparing DT and HT conditions with YP. GWAS detected common genomic regions and 201 MTAs for the stress indices (Tables S8, S9).

### Drought stress indices

The number of MTAs detected for YLD-SSI, TGW-SSI, and GNO-SSI were 7, 11, and 13, respectively. For TOL, 142 MTAs were detected, while 28 MTAs were detected for STI under DT (Table S8). A comparison of these indicated several common regions (Table 6). Among them, loci on chromosomes 2B (83 cM), 3A (48 cM), 3B (50 cM and 113 cM), 5A (150 cM), 5B (101 cM), 6A (28 cM), 6B (31 cM), and 7A (75 cM) were associated with multiple stress indices. The most common loci associated with DT indices were on chromosomes 2B (83 cM), 3A (48 cM), 3B (50 cM), 5A (20–22 and 150 cM), 6A (28 cM), and 6B (31 cM) (Table 6 and Figure S4).

**Table 6:** Comparison of GWAS results for drought stress indices.

**Chr**.	**Traits**								
	**YLD**			**GNO**			**TGW**		
	**SSI**	**TOL**	**STI**	**SSI**	**TOL**	**STI**	**SSI**	**TOL**	**STI**
1A	–	–	–	–	–	–	77	77, 81, 143	–
1B	–	–	–	–	–	–	–	51, 55, 160, 172	101
2A	–	–	–	123	63–66	123	75, 87	68–76, 87–88, 104–123	–
2B	83	–	83	82–83	–	82–83	12	12, 18, 22, 76–85	–
3A	48	–	48	48	48	48	–	4, 40, 48, 66	–
3B	–	50, 113	–	50, 85, 112	112	50, 85, 112	45	50, 79–82	45
4A	–	–	–	–	–	–	96	96, 132	–
4B	–	–	–	–	39–42	–	–	33, 46, 47	–
5A	150	20, 22	150	150	–	–	36, 86	13, 35–38, 50, 57, 68, 84, 86, 111, 115, 150	84–86
5B	19, 101	–	19, 101	–	–	–	–	32, 48	–
6A	28	–	28	28, 50	48–52, 82, 94	–	91	55, 59	91, 98
6B	31	–	31	31	0–4	–	78	56, 82	78
7A	–	75	–	–	–	–	140	67, 73, 75, 140, 157	–
7B	–	–	–	–	–	–	–	36, 39, 40, 47–48, 92, 128	–

*For each trait, the genetic position is represented by numbers. YLD, grain yield (t/ha); TGW, thousand grain weight (g), GNO; grain number/m^2^; SSI, stress susceptibility index; TOL, stress tolerance; STI, stress tolerance index*.

### Heat stress indices

We identified 212 MTAs for HT indices (Table S9), with the highest number of MTAs on chromosomes 1B and 2A. The most common loci associated with HT indices for YLD were on chromosomes 2B (74–85 cM), 3B (68–83 cM), 4A (107–124 cM), and 7A (24 cM) (Figure S5 and Table 7). Examples of the effect of the most common MTAs for traits and stress indices are shown in Figures S7, S8.

**Table 7:** Comparison of GWAS results for heat stress indices.

**Chr**.	**Traits**								
	**YLD**			**GNO**			**TGW**		
	**SSI**	**TOL**	**STI**	**SSI**	**TOL**	**STI**	**SSI**	**TOL**	**STI**
1A	–	–	–	–	–	–	–	–	–
1B	142	–	–	160–171, 281	144, 160–161	160–172	51, 84–85, 101, 197	197	51, 84–85, 101, 197
2A	58	–	–	–	54–72	–	64–69	45, 62–69	64–69
2B	63, 74	–	–	76	–	76	–	75, 82	–
3A		–	–	–	–	–	30	–	30
3B	49, 68, 77–83	39	78–83	11, 77–83	68, 116	11, 77–83	–	–	–
4A	124	–	107, 124	–	–	124	124	–	–
4B	32–33	–	–	33	–	–	–	–	–
5A	30	–	–	–	–	–	84	35, 63	35, 84
5B	26	32, 61	–	55	76	55	–	–	–
6A	58	–	–	–	–	–	74, 91	91	74, 91
6B	31	–	–	–	13	13	21–22, 33	21–22, 33	21–22, 33
7A	96	25	25	25	25	25	–	–	–
7B	–	–	–	–	–	–	–	94	–

*For each trait, the genetic position is represented by numbers. YLD, grain yield (t/ha); TGW, thousand grain weight (g), GNO; grain number/m^2^; SSI, stress susceptibility index; TOL, stress tolerance; STI, stress tolerance index*.

### Markers for traits under irrigation (YP and HT combined), stressed environments (DT and HT combined), and combined analysis of all three environments (YP, DT, and HT)

**Figure 5:**
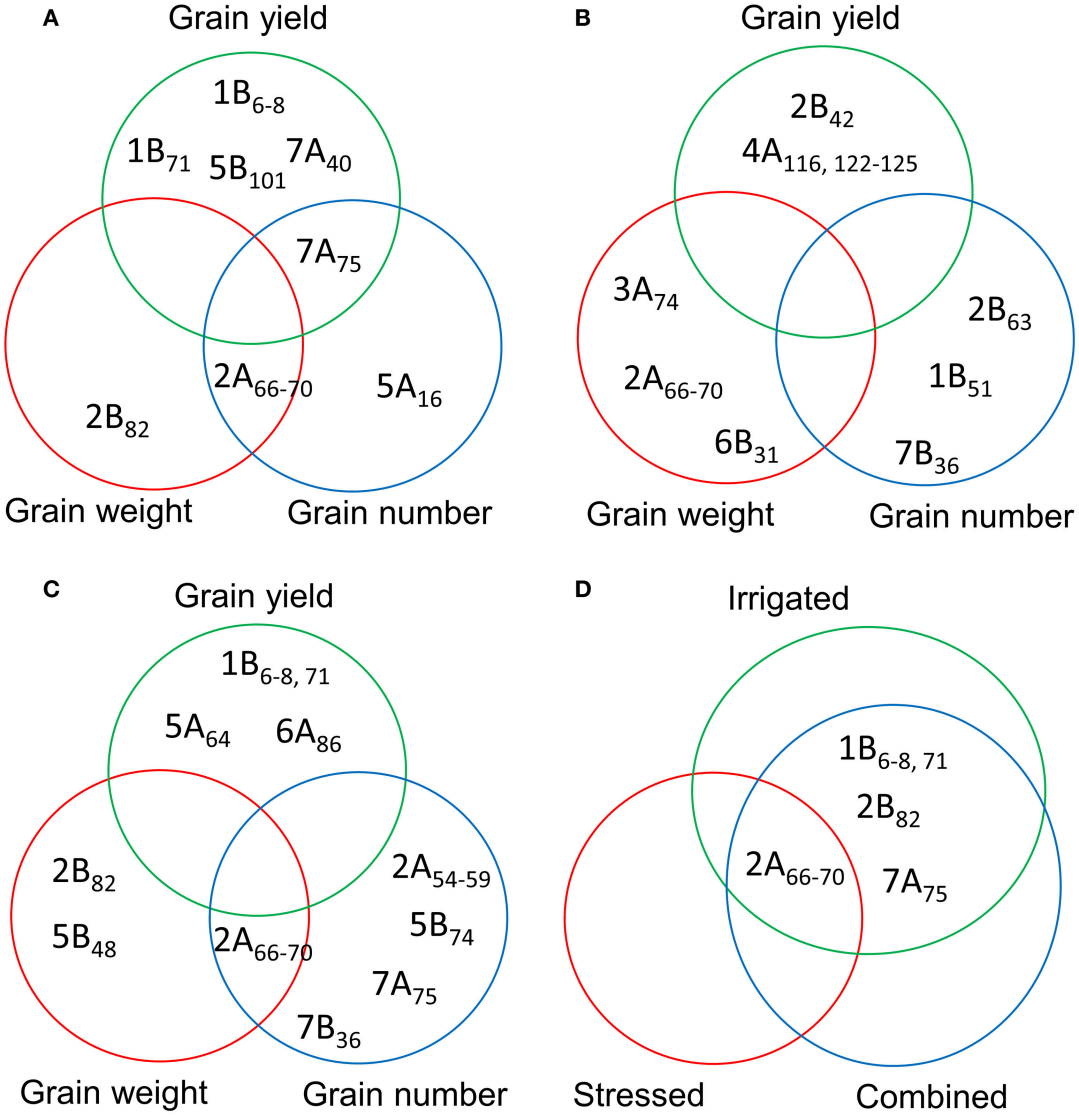
Comparison of the MTAs for grain yield, grain number, and thousand grain weight based on the combined analysis of **(A)** irrigated (yield potential and heat stress); **(B)** stressed (drought and heat stress); and **(C)** combined analysis of the three environments for the traits; and **(D)** common MTAs detected by comparing the three different analyses **(A–C)**.

We detected 81 MTAs under irrigated conditions (combined analysis of YP and HT conditions). Of these, a locus on chromosome 2A (66–70 cM) was associated with TGW (independent of DTA), while a locus on chromosome 2B (65–75 cM) was associated with DTA, DTM, PH, and TGW (Figure 5A). Markers for YLD were identified on chromosomes 1B (6 and 8 cM), 5B (101 cM), and 7A (40 and 75 cM). There were 23 markers associated with DTA, mostly located on chromosomes 2A, 2B, and 3A. We detected 19 MTAs for PH, the most significant of which was on chromosome 4B (31 cM) (Table S10). Under irrigated conditions, a common locus for TGW and GNO was identified on chromosome 2A (66–70 cM), and for YLD and GNO on chromosome 7A (75 cM) (Figure 5A). No common locus was identified for YLD, TGW, and GNO. Four loci on chromosomes 1B (6–8 and 71 cM), 5B (101 cM), and 7A (40 cM) were associated with YLD *per se*, but were not associated with GNO or TGW (Table S10).

We detected 93 MTAs for the traits in stressed environments (combined BLUPs of DT and HT conditions). Chromosome 4A (122–125 cM) harbored the highest number of MTAs for YLD. For TGW, the highest number of associations was on chromosome 2A (66–70 cM). As with irrigated conditions, most of MTAs identified for PH were on chromosome 4B (31–32 cM) (Table S11). No common locus for YLD, TGW, and GNO was identified in this analysis (Figure 5B). A comparison of MTAs detected by the combined BLUPs of three different environments indicated the most consistent locus for TGW and GNO on chromosome 2A (66–70 cM) (Figure 5C and Figure S6). The same locus was associated with many other traits (Table S12). Two loci on chromosomes 2B (82 cM) and 5B (48 cM) were associated with TGW, but not with YLD. Similarly, loci on four chromosomes (2A, 5B, 7A, and 7B) were identified for GNO but not YLD (Figure 5C).

A comparison between the MTAs under irrigated, stressed, and combined analysis of three environments indicated a locus on chromosome 2A (66–70 cM) as the most common. Loci common to irrigated and combined analyses were identified on chromosomes 1B (6–8 cM and 71 cM) for YLD, 2B (82 cM) for TGW, and 7A (75 cM) for GNO (Figure 5D).

### QTL hotspots for YLD, GNO, TGW, and flowering time

**Figure 6:**
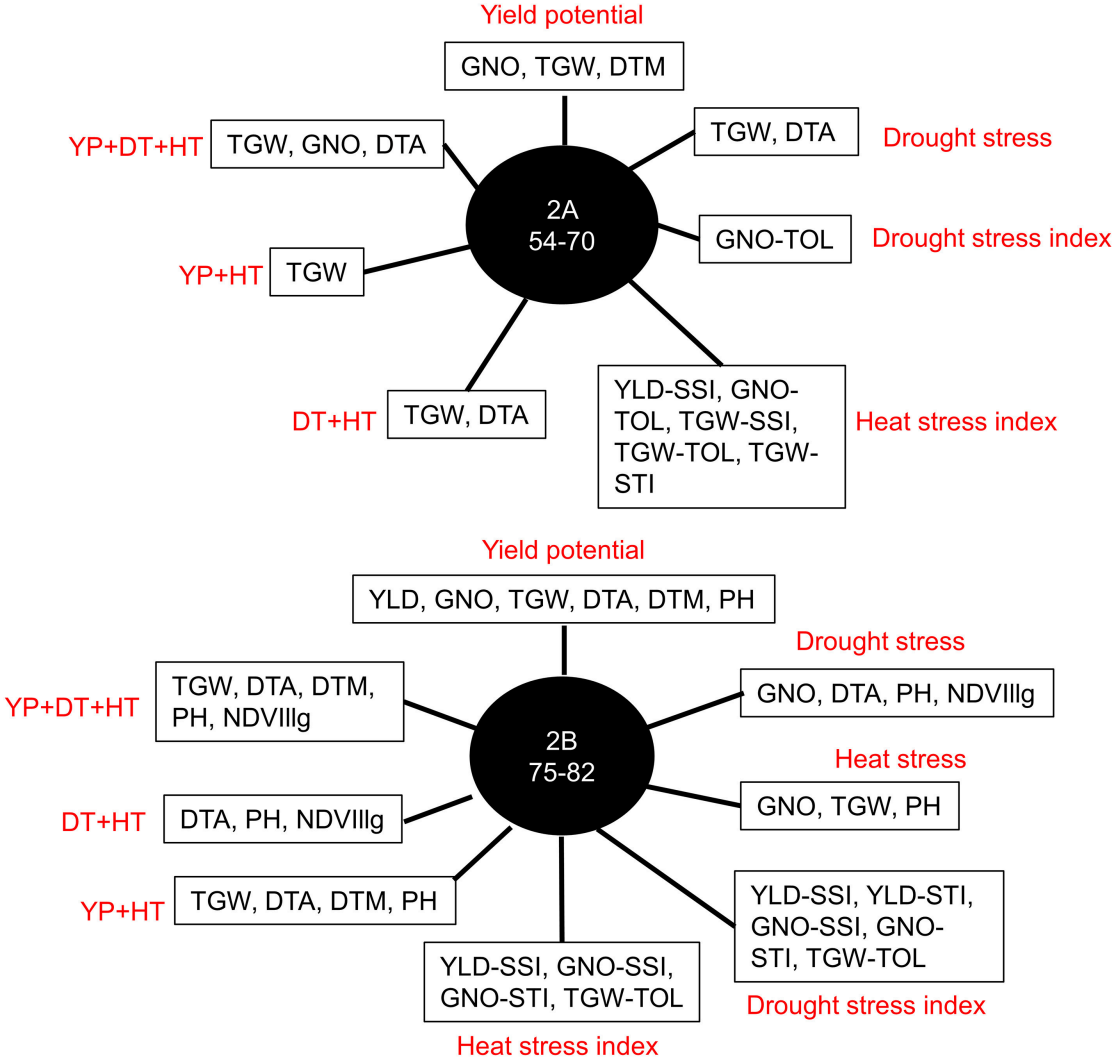
Most common markers associated with multiple traits in different environments: yield potential (YP), drought stress (DT), and heat stress (HT); stress indices (SSI, TOL, and STI); and combined analyses of irrigated (YP+HT), stressed (DT+HT), and combined analyses of irrigated and stressed environments (YP+DT+HT).

**Figure 7:**
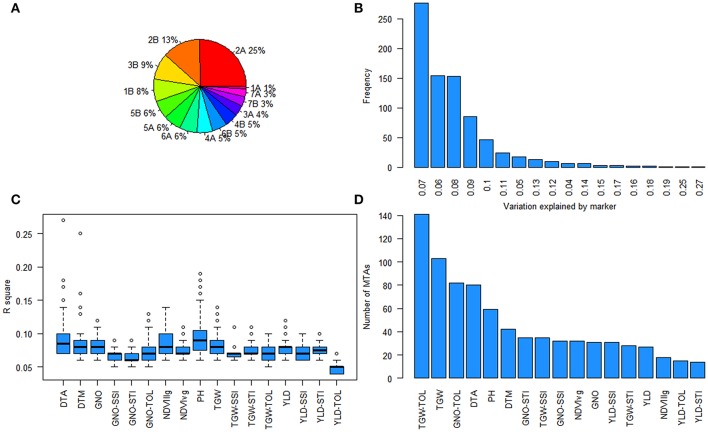
Summary of marker-trait associations (MTAs) **(A)** Pie chart showing the percentage of MTAs in different chromosomes; **(B)** frequency of the variation explained by the MTAs; **(C)** the range of percentage of variation explained for each trait; and **(D)** number of MTAs for each trait.

Further analysis of the GWAS results indicated two loci affecting YLD, TGW, and GNO in different environments (Table S13). Loci on chromosomes 2A (54–70 cM) and 2B (75–82 cM) affected multiple traits, including stress indices (Figure 6). The highest number of MTAs were detected on chromosome 2A (25%) followed by 2B (13%), with the least on chromosome 1A (1%) (Figure 7A). The variation explained by the markers varied from 7 to 27% (Figure 7B). DTA had markers explaining the highest variation (>25%), followed by DTM (>24%), and PH (close to 20%) (Figure 7C). The highest number of MTAs were detected for TGW-TOL combining all stress environments— under YP, DT, and HT environments—followed by TGW and the lowest number of MTAs were detected for YLD tolerance indices estimated as TOL and STI (Figure 7D).

### Candidate genes and syntenic regions

We further analyzed the SNPs in chromosomes 2A (54–70 cM) and 2B (75–82 cM) that were identified as QTL hotspots. There were 28 and 16 unique significant SNPs observed in the 2A and 2B region, respectively. BLASTN analysis of the SNP sequences on Ensemble genome browser for wheat and barley genomes indicated that several SNPs were related to transmembranes or were uncharacterized proteins. Of these, one SNP (100035706) was related to the gene *DMAS1-A* with a protein characterized as *Deoxymugineic acid synthase 1*. The physical location of this gene was in chromosome 4AS in bread wheat and in chromosome 4H in barley. In some cases, the hits were on homeologous chromosomes 2A, 2B, and 2D in bread wheat and it were observed in different chromosomes in Barley. We also BLASTed the SNP sequences against the UGRI durum Capelli v1 and Durum Strongfield v1, but the results were on chromosome contigs without further annotation.

## Discussion

This study evaluated a durum wheat panel systematically assembled from the CIMMYT gene bank for YLD, TGW, GNO, DTA, DTM, PH, NDVIvg, and NDVIlg under YP, DT, and HT conditions. The panel was phenotyped by adjusting planting dates to create YP, DT, and HT conditions in the field. Under HT, mean YLD of the panel reduced by 72%, which was larger than the reduction in YLD under DT (60%), when compared with YP conditions. Similar trends were previously reported in a study on a bi-parental population in similar environments (Pinto et al., 2010). In our study, HT was independent of DT, as irrigation was supplied under HT (similar to yield potential).

We observed moderate to high H^2^ estimates for the traits, which is similar to the results achieved by Sukumaran et al. (2014) when they observed spring bread wheat under similar management conditions in Cd. Obregon, Mexico. YLD was negatively associated with DTA and DTM under YP, DT, and HT conditions, indicating a yield advantage for earlier genotypes (Millet et al., 2016). An important question now is whether grain-filling duration affects YLD under these conditions in durum wheat, as earlier studies have shown that grain filling duration and rate is reduced and starch synthesis inhibited in spring wheat when temperatures are above 30°C (Reynolds et al., 2012). In our study, we did not observe association between grain filling duration and grain yield or TGW when different stress conditions and YP were compared. Another phenology trait, PH, was negatively associated with YLD under YP, but no significant association was observed under DT, and PH was positively associated with YLD under HT. This positive association of PH and YLD under HT may be related to higher biomass of the plants; as biomass and PH are positively associated (Reynolds et al., 2017).

NDVI was highly associated with YLD under HT, indicating that it is an effective selection tool for in-season prediction of wheat YLD (Tattaris et al., 2016). GNO was highly associated (>0.72) with YLD in all environments, whereas TGW was more significantly associated with YLD under HT than under YP and DT. This indicates that there is a tradeoff between TGW, GNO, and other yield components under varying environment conditions and further research is needed (Sukumaran et al., 2017).

GWAS is the most popular approach for dissecting the genetic basis of complex traits (Risch and Merikangas, 2007), but this approach is prone to the detection of false positives due to confounding population structure or the effect of phenology genes (Yu et al., 2006; Zhang et al., 2010). Our study used the mixed model framework of Yu et al. (2006), with fixed and random effects to control false positives. Q-Q plots for multiple models were evaluated to select the best models for identifying MTAs (Sukumaran et al., 2014). We also observed DTA and DTH were associated with grain yield in this panel. Using phenology as a covariate did not remove the co-localization of QTLs for flowering time and agronomic traits, indicating the strong association between these traits. GWAS was performed with BLUP values estimated with and without DTA/DTH as a covariate. We identified several MTAs for YLD and component traits co-localized with and without phenology genes. Flowering time and PH are associated with adaptation and agronomic performance of traits in several crops, and can be related to drought escape (Shavrukov et al., 2017). This is observed not only in wheat, fine tuning flowering time though earliness *per se* (*Eps*) genes (Zikhali et al., 2014, 2016; Sukumaran et al., 2016), but also in barley, controlled by the *EPS2/Eam6* gene with pleotropic effects on agronomic traits (Tondelli et al., 2014). Earlier studies on barley have reported that YLD is defined by the length of different sub-phases of vegetative, flowering, and grain filling stages (Francia et al., 2011).

Co-localizing MTAs were identified for YLD, GNO, and TGW, and individual MTAs were observed for each trait. We also identified MTAs for YLD that were independent of phenology (on chromosomes 6A at 34 cM for YLD under YP and 5B at 30 and 60 cM for YLD under DT). This indicates that—in addition to GNO and TGW—other yield components (e.g., plant density) are also important for increasing YLD (Sukumaran et al., 2015). In addition, the individual traits TGW and GNO can be manipulated, independently of YLD, as unique MTAs were observed for them under different conditions (Sukumaran et al., 2017). We identified many genomic regions associated with the traits; some of them—on chromosomes 2A and 2B—were common to both YP and DT conditions. As far as we know, this is the first comprehensive study focusing on MTAs and their interactions for YLD and its components under YP, DT, and HT conditions in durum wheat. There were fewer MTAs for NDVI, and some co-located with the agronomic traits, though a comparison with an earlier study on nitrogen use efficiency indicated that common QTLs for NDVI may exist on chromosome 3B (Quraishi et al., 2011; Monostori et al., 2017). An earlier study on durum wheat using 10 rainfed and 6 irrigated environments also identified a major YLD QTL on chromosome 2B with significant effects (Maccaferri et al., 2008). Another study used an association mapping approach in durum wheat and identified markers for YLD under different drought stress conditions (Maccaferri et al., 2011). A common genetic map could be used to compare these results with our own and identify common loci.

Common genetic markers for TGW and GNO were detected on chromosomes 2A and 2B under DT and YP conditions but, under HT, the markers associated with TGW and GNO were on chromosome 4B. This indicates that the genetic basis for YLD, TGW, and GNO were determined through a different mechanism in HT, compared to DT and YP conditions. An evolutionary study in wheat comparing wild emmer (*T. turgidum* subsp. *dicoccoides*) and durum wheat for TGW and embryo size identified a cluster of loci affecting TGW and grain shape in the long arm of chromosome 2AL, and a novel locus controlling embryo weight in chromosome 2AS (Golan et al., 2015). A BLAST search of the 2A and 2B hotspots indicated that they might be homeologous QTLs, as several SNP hits were observed in chromosomes 2A and 2B. The *HvCEN* gene with pleotropic effects on flowering time and agronomic traits previously found in chromosome 2H of barley (Francia et al., 2011; Comadran et al., 2012; Tondelli et al., 2014) might be syntenic to the 2A hot spot region (BLAST hits on 2AL:32101-32658; TGACv1 for bread wheat). We detected more MTAs for GNO under DT, though the number of markers for TGW were higher in HT.

Breeding for wide adaption is hindered by high genotype × environment interactions. In this study, we identified loci stable across DT and HT environments using three different stress indices. These stress indices had several common MTAs, which were also associated with YLD and its components. Further studies are required to understand and validate the effect of these markers. The markers on chromosomes 2B (83 cM), 3B (50 cM), 5A (150 cM), 6A (28 cM), and 6B (31 cM) are candidates for further exploration of drought stress tolerance. The loci on chromosomes 2B (74–83 cM) and 3B (68–83 cM) are prominent loci for heat stress tolerance, which may be similar to an earlier study for drought stress (Maccaferri et al., 2008).

More powerful statistical genetics tools would be needed to detect minor alleles associated with the traits. The panel used in this study had a group of lines from West Asia and North Africa, which was genetically different from other entries in the panel, but the low number of lines does not allow for identification of MTAs on minor alleles, if present. Pleiotropy is another aspect of the study where several traits were associated with QTL hotspots in chromosomes 2A and 2B. The locus on chromosome 2A (54–70 cM) affects multiple traits and stress indices indicating QTL hotspots (Figures S7, S8). This QTL region might need to be recombined by mutation or TILLING to see the effect of MTAs on individual traits (Uauy et al., 2009). The variation explained by the MTAs varied from 7 to 27%, indicating that SNP-based GWAS may need to be complemented by haplotype-based GWAS to explain the missing variation (N'Diaye et al., 2017). However, accurate detection of haplotypes based on general LD decay may not give promising results and may need to be substituted by LD block-based haplotypes.

This study contributes large number of MTAs in durum wheat for agronomic traits under YP, DT, and HT conditions. Stable loci across environments were identified that can be further explored for use in marker-assisted selection and gene discovery. Plants' responses to abiotic stresses are complex and it is essential to identify regulatory loci if these traits are to be manipulated. Climate change is predicted to have significant impacts in the Mediterranean region. To mitigate climate change, genomic technologies, and resources with genetic approaches need to be coupled through marker-assisted selection (Habash et al., 2009). Instead of conventional direct selection for YLD, genetic loci for yield components and associated traits should be identified to enable the manipulation of individual traits to obtain the cumulative gene action for enhancing genetic gains in wheat (Reynolds et al., 2009a; Reynolds and Langridge, 2016). Additionally, the recent availability of high-quality genome assemblies for tetraploid wheat (Avni et al., 2017), coupled with the analysis of transcriptome profiles under DT conditions (Habash et al., 2014), will facilitate the identification and manipulation of causative loci governing yield stability across a broad range of environmental conditions.

## Author contributions

SS and MR: Conceived and designed the study; SS and CS: Genotyped the panel; CS: Aligned the markers on the consensus map and reference genome; SS: Did the genetic and phenotypic analyses; SS: Wrote the manuscript; All authors reviewed the manuscript.

### Conflict of interest statement

The authors declare that the research was conducted in the absence of any commercial or financial relationships that could be construed as a potential conflict of interest.

## Supplementary material

Figure S1 Results for population structure indicating five sub-populations in the durum panel, based on Evanno et al. (2005).

Figure S2 LD decay plots of the durum panel considering all chromosomes indicated the LD decay between 5 and 10 cM.

Figure S3 Correlations between the stress indices (SSI, TOL, and STI) for grain yield (YLD), grain number (GNO), and grain weight (TGW) under yield potential (YP), drought stress (DT), and heat stress (HT) conditions.

Figure S4 Manhattan plots and quantile-quantile plots for the stress indices **(A)** SSI, **(B)** TOL, and **(C)** STI for grain yield comparing yield potential and drought stress conditions. Numbers in the Manhattan plots indicate the chromosome position of the most common significant marker-trait associations.

Figure S5 Manhattan plots and quantile-quantile plots for the stress indices **(A)** SSI, **(B)** TOL, and **(C)** STI for grain yield comparing yield potential and heat stress conditions. Numbers in the Manhattan plots indicate the chromosome position of the most common significant marker-trait associations.

Figure S6 Comparison of the MTAs under **(A)** Irrigated (yield potential and heat stress); **(B)** stressed (drought and heat stress); and **(C)** combined analysis of the three environments for the highest heritability trait; thousand-grain weight indicating common MTAs in chromosome 2A (54–70 cM) and 2B (75–82 cM).

Figure S7 The effect of the 2A marker at 54–70 cM on traits under **(A)** Irrigated yield potential; **(B)** drought stress; and **(C)** heat stress conditions.

Figure S8 The effect of the 2A marker on stress indices (SSI, TOL, and STI) for **(A)** Grain yield; **(B)** thousand-grain weight; and **(C)** grain number.

Table S1 Description of the entries from the durum wheat panel.

Table S2 GWAS results for irrigated environments for all traits.

Table S3 GWAS results for the traits under yield potential conditions using flowering time as a covariate.

Table S4 GWAS results for drought stressed environments for all traits.

Table S5 GWAS results for the traits under drought stress conditions using flowering time as a covariate.

Table S6 GWAS results for heat stressed environments for all traits.

Table S7 GWAS results for the traits under heat stress conditions using flowering time as a covariate.

Table S8 GWAS results for SSI, TOL, STI for drought stress.

Table S9 GWAS results for SSI, TOL, STI for heat stress.

Table S10 GWAS results for the combined analysis of irrigated conditions (yield potential and heat stress).

Table S11 GWAS results for the combined analysis of stressed environments (drought and heat stress).

Table S12 GWAS results of the combined analysis of the traits from yield potential, heat stress, and drought stress.

Table S13 Comparison between the MTAs identified for yield and yield components with that of stress indices.

## Abbreviations

BLUP: best linear unbiased prediction
DTA: days to anthesis
DTH: days to heading
DTM: days to maturity
GNO: grain number m^−2^
GWAS: genome-wide association study
IWIN: International Wheat Improvement Network
IWIS: International Wheat Information System
LD: linkage disequilibrium
MTA: marker-trait association
NDVI: normalized difference vegetative index
NDVIllg: NDVI at grain filling stage
NDVIvg: NDVI at vegetative stage
PH: plant height
SSI: stress susceptibility index
TOL: stress tolerance
SNP: single nucleotide polymorphism
STI: stress tolerance index
TGW: thousand grain weight
YLD: grain yield.
