# Supplementary figures and images for: Genome-Wide Association Analyses Identify QTL Hotspots for Yield and Component Traits in Durum Wheat Grown under Yield Potential, Drought, and Heat Stress Environments
Source: Front Plant Sci. 2018 Feb 6;9:81. doi: 10.3389/fpls.2018.00081 (PMC5808252; doi:10.3389/fpls.2018.00081)

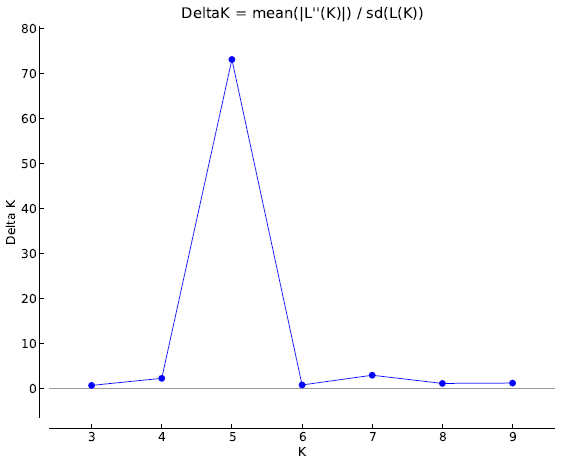

Supplement: Figure S1 — Results for population structure indicating five sub-populations in the durum panel, based on Evanno et al. (2005). [file Image1.TIF]

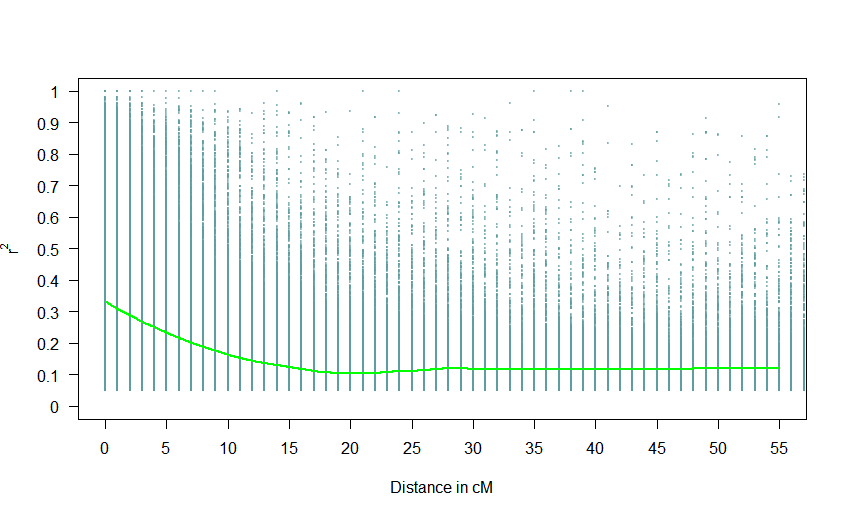

Supplement: Figure S2 — LD decay plots of the durum panel considering all chromosomes indicated the LD decay between 5 and 10 cM. [file Image2.TIFF]

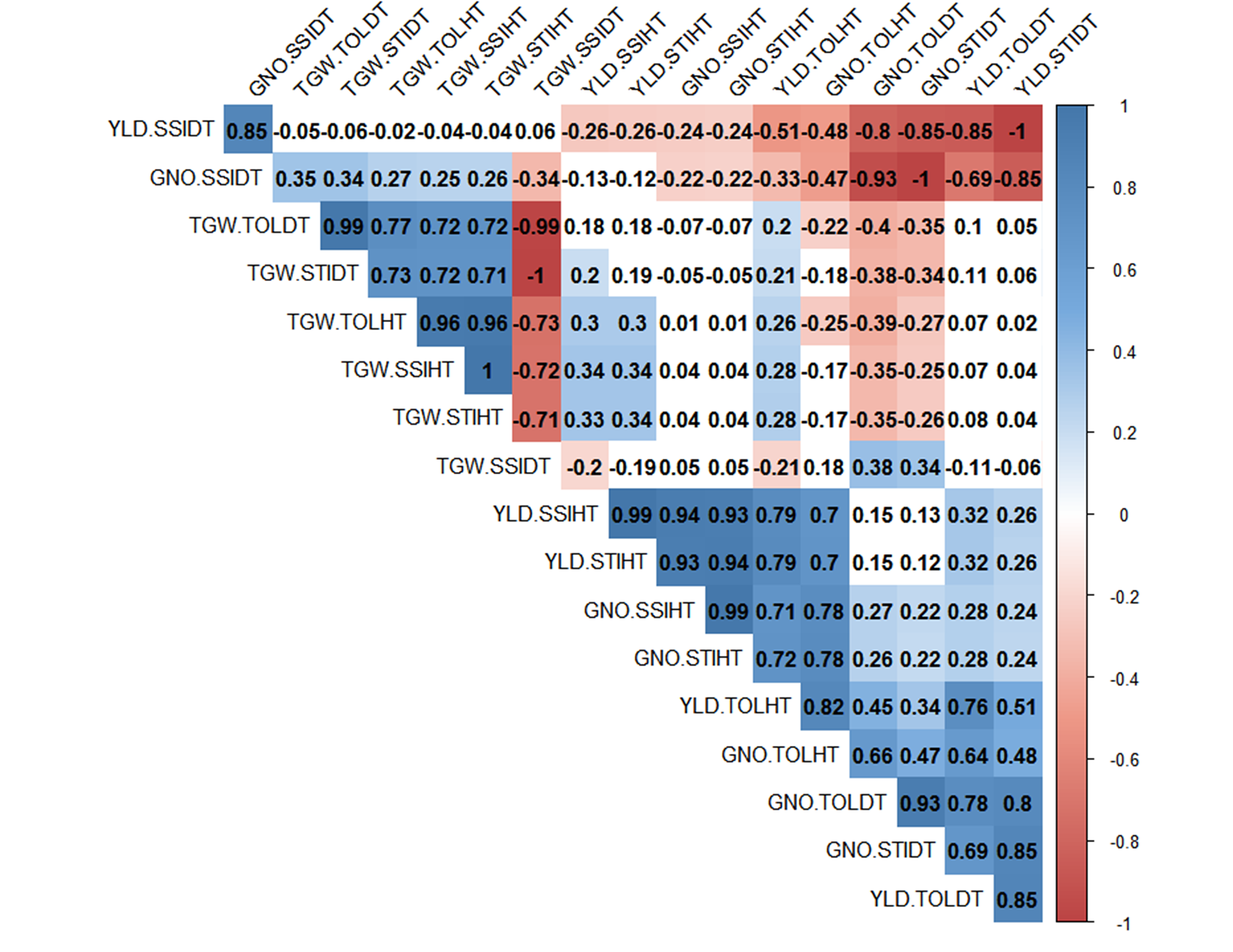

Supplement: Figure S3 — Correlations between the stress indices (SSI, TOL, and STI) for grain yield (YLD), grain number (GNO), and grain weight (TGW) under yield potential (YP), drought stress (DT), and heat stress (HT) conditions. [file Image3.TIF]

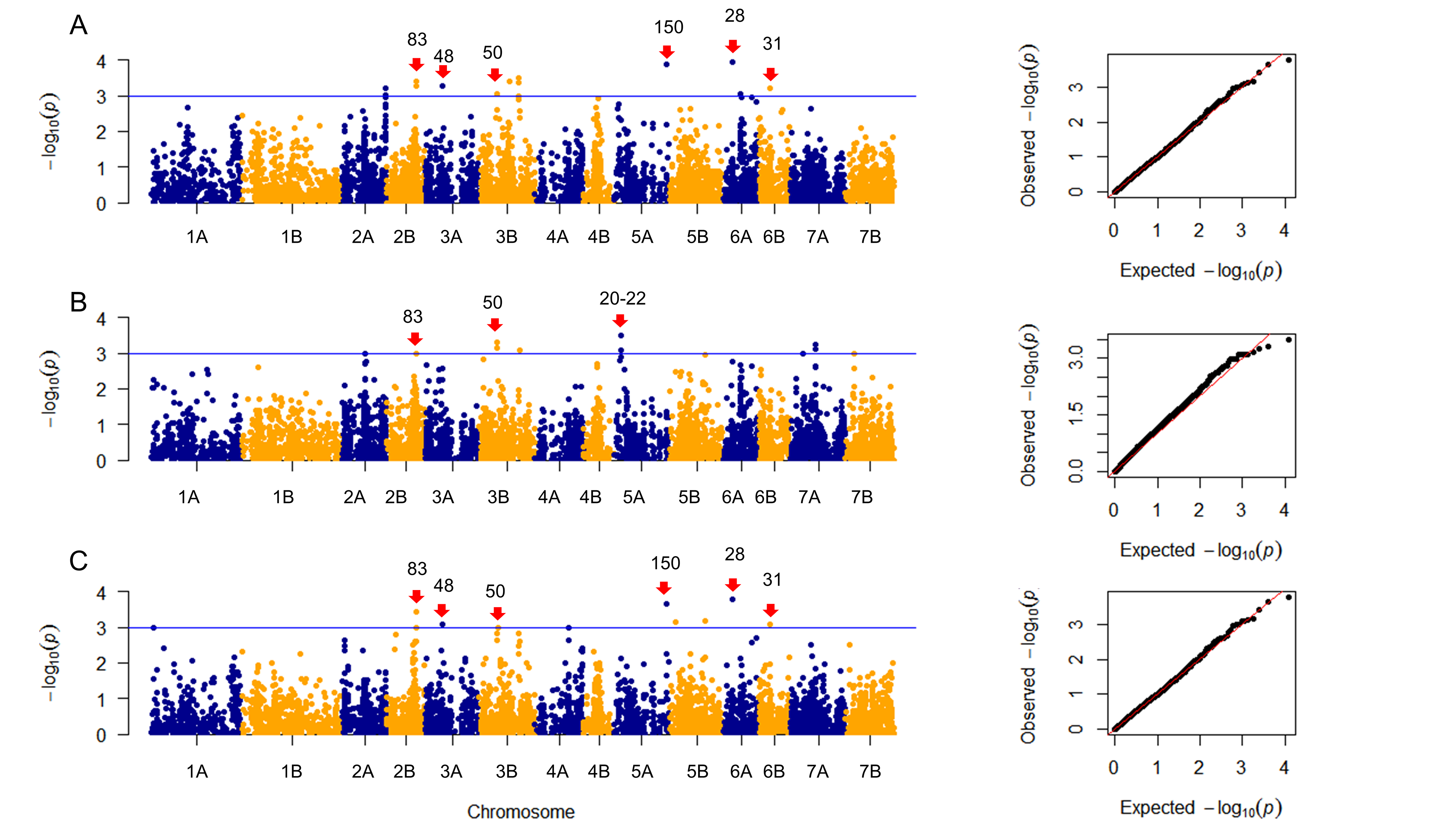

Supplement: Figure S4 — Manhattan plots and quantile-quantile plots for the stress indices (A) SSI, (B) TOL, and (C) STI for grain yield comparing yield potential and drought stress conditions. Numbers in the Manhattan plots indicate the chromosome position of the most common significant marker-trait associations. [file Image4.TIF]

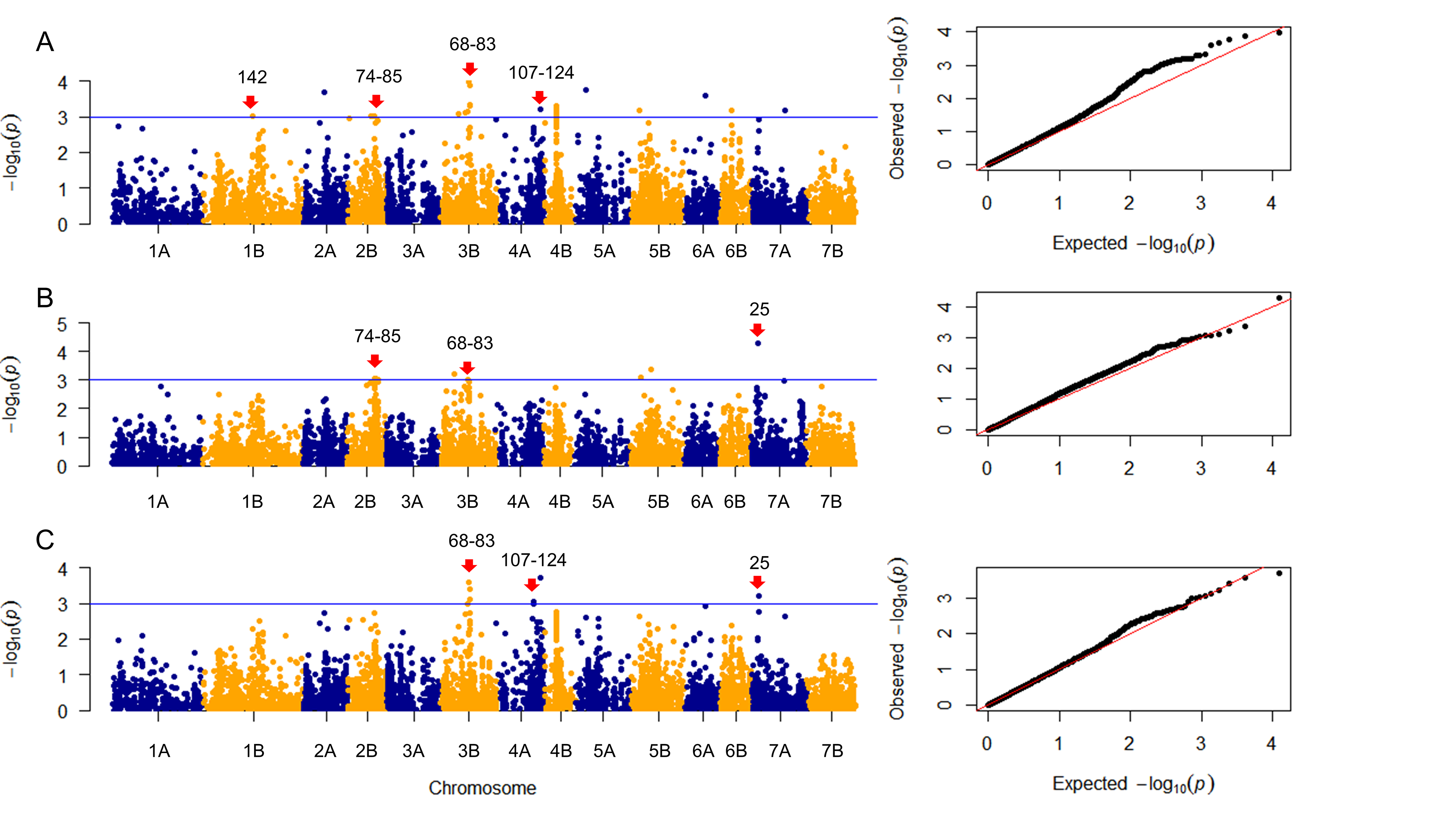

Supplement: Figure S5 — Manhattan plots and quantile-quantile plots for the stress indices (A) SSI, (B) TOL, and (C) STI for grain yield comparing yield potential and heat stress conditions. Numbers in the Manhattan plots indicate the chromosome position of the most common significant marker-trait associations. [file Image5.TIF]

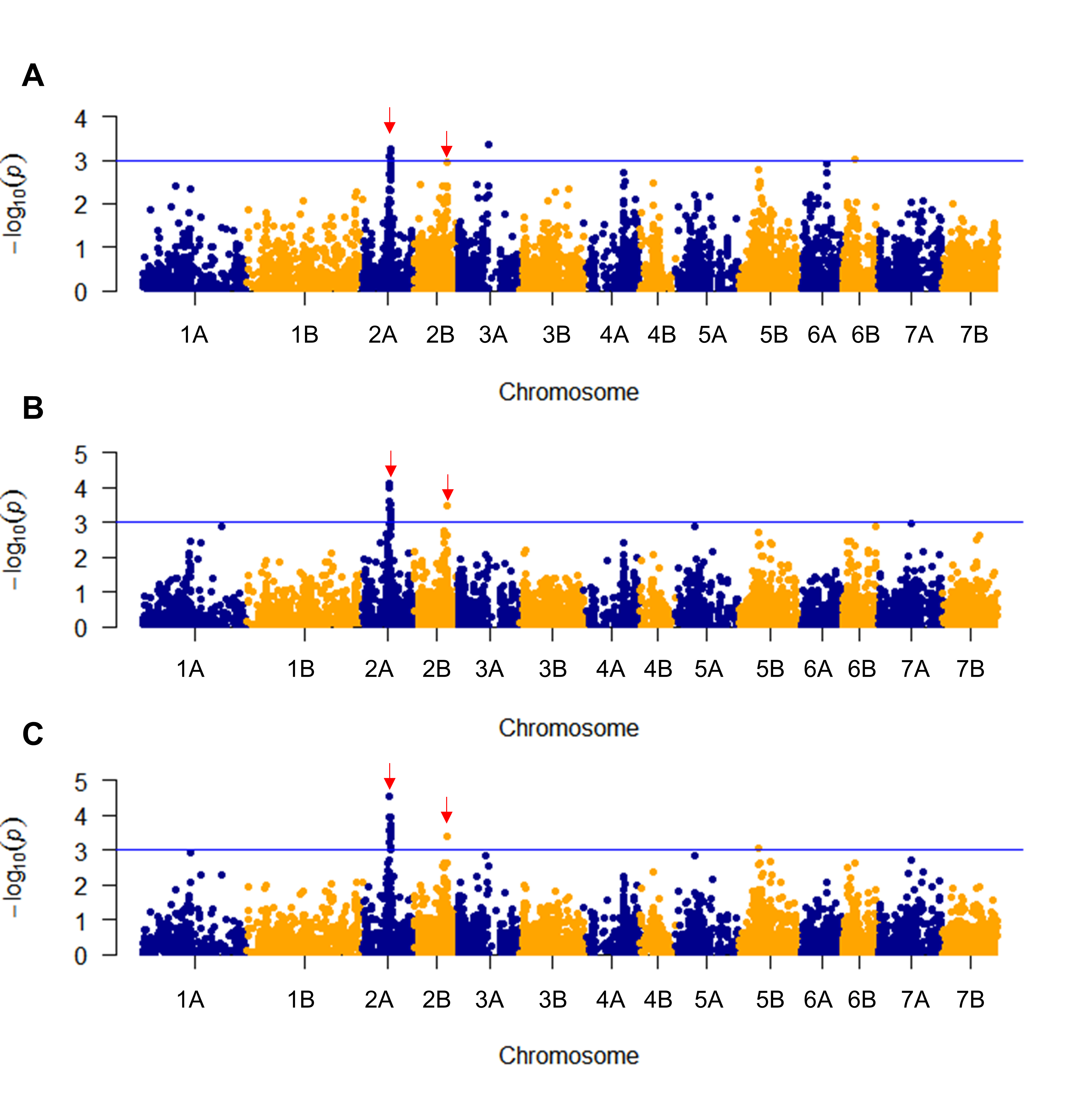

Supplement: Figure S6 — Comparison of the MTAs under (A) Irrigated (yield potential and heat stress); (B) stressed (drought and heat stress); and (C) combined analysis of the three environments for the highest heritability trait; thousand-grain weight indicating common MTAs in chromosome 2A (54–70 cM) and 2B (75–82 cM). [file Image6.TIF]

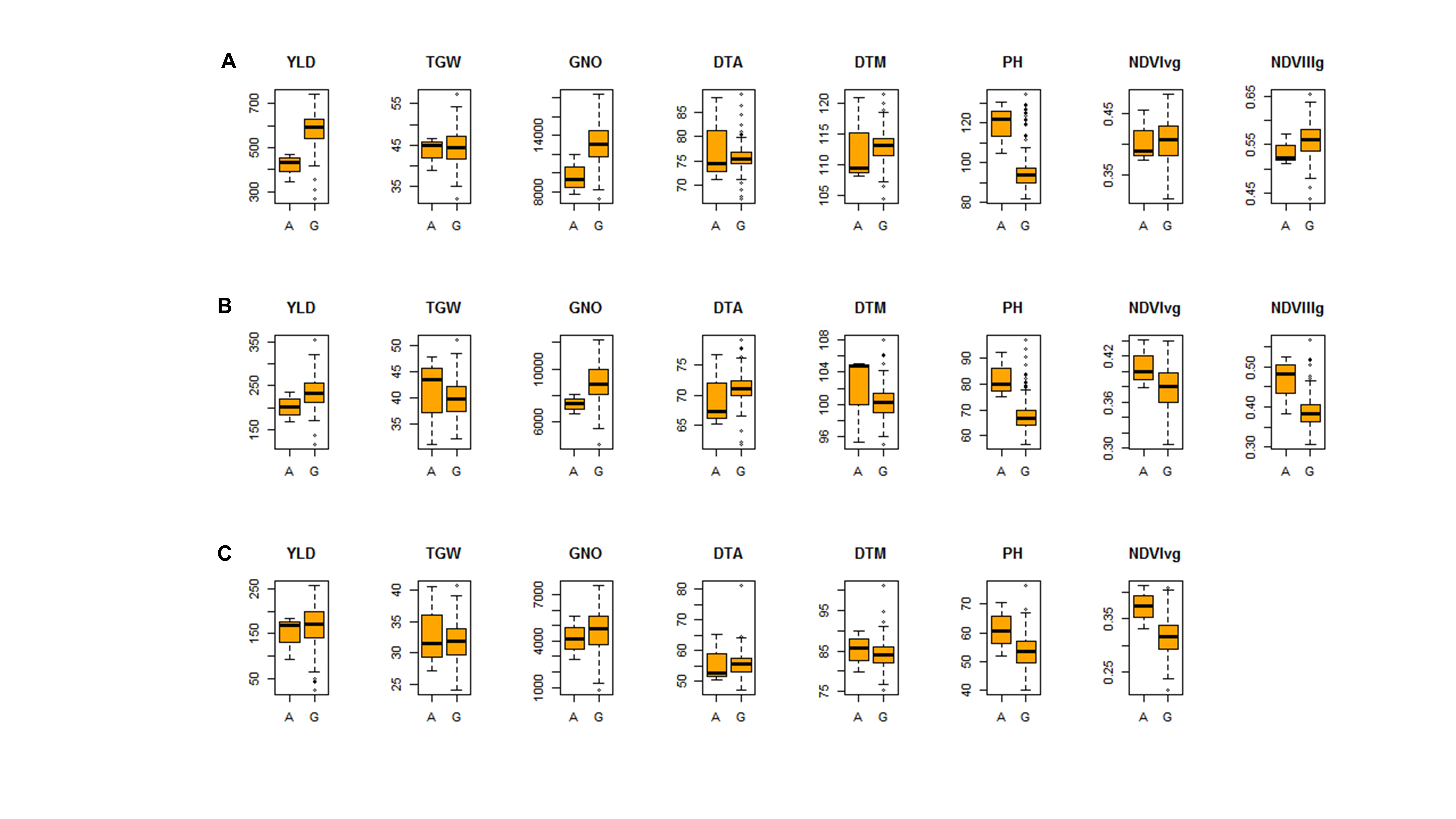

Supplement: Figure S7 — The effect of the 2A marker at 54–70 cM on traits under (A) Irrigated yield potential; (B) drought stress; and (C) heat stress conditions. [file Image7.TIF]

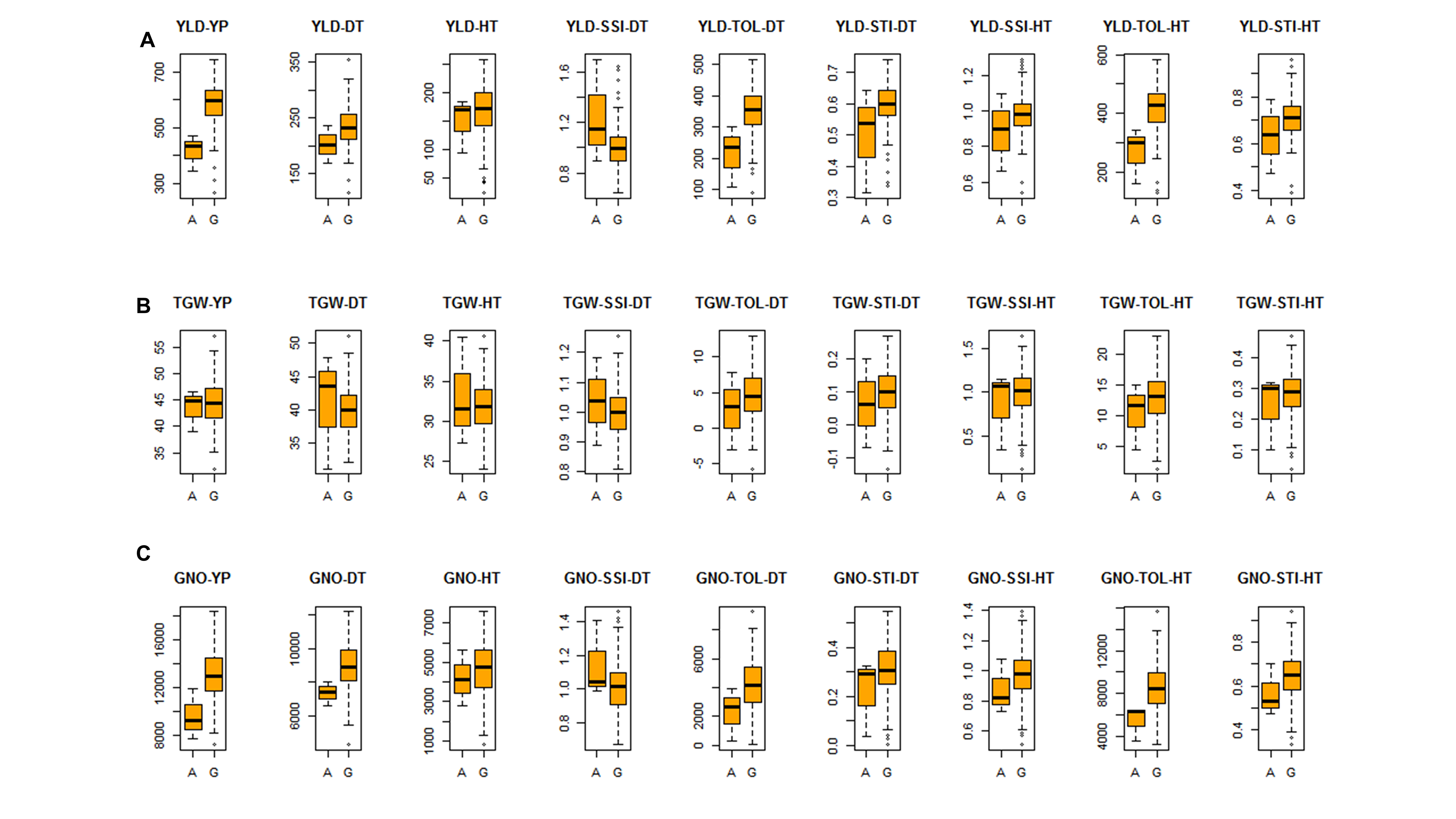

Supplement: Figure S8 — The effect of the 2A marker on stress indices (SSI, TOL, and STI) for (A) Grain yield; (B) thousand-grain weight; and (C) grain number. [file Image8.TIF]
